# Meningococcal Carriage among Household Contacts of Patients with Invasive Meningococcal Disease in Kathmandu, Nepal: A Longitudinal Study

**DOI:** 10.3390/pathogens10070781

**Published:** 2021-06-22

**Authors:** Supriya Sharma, Jyoti Acharya, Dominique A. Caugant, Shreedhar Aryal, Megha Raj Banjara, Prakash Ghimire, Anjana Singh

**Affiliations:** 1 Central Department of Microbiology, Tribhuvan University, Kirtipur, Kathmandu 44600, Nepal; megha.banjara@cdmi.tu.edu.np (M.R.B.); prakash.ghimire@cdmi.tu.edu.np (P.G.); anjana.singh@cdmi.tu.edu.np (A.S.); 2 National Public Health Laboratory, Teku, Kathmandu 44600, Nepal; jyotigan30@gmail.com; 3 WHO Collaborating Centre for Reference and Research on Meningococci, Norwegian Institute of Public Health, 0213 Oslo, Norway; dominiqueandreeyvette.caugant@fhi.no; 4 Bhaktapur Hospital, Dudhpati, Bhaktapur 44800, Nepal; helloshreedhar@gmail.com

**Keywords:** meningococci, carriage, duration, serogroup, Nepal

## Abstract

Because asymptomatic carriers are key source of transmission, information on meningococcal carriage in the community provides a scientific basis for appropriate preventive/control strategies. This longitudinal study (January 2017–December 2019) aimed to estimate carriage rate of meningococci among household contacts of meningococcal meningitis cases within Kathmandu Valley, Nepal. Throat swab samples were collected at first visit from each person in households, twice a month for up to 2 months and subsequently on a monthly basis for a further 4 months. Altogether, 1125 throat samples were processed by conventional culture for the identification of meningococci. To the best of our knowledge, this is the first longitudinal study on meningococcal carriage in Nepal. The meningococcal carriage rate among household contacts was 15%. All carriers were aged 19 years or older. There was no statistically significant gender difference. The duration of carriage was 60 days. Twenty of 36 isolates belonged to serogroup A, and 16 were non-serogroupable (NG). Serogroups isolated from the same individuals did not change within the follow-up period. All meningococcal isolates over the past 38 years in Nepal that have been reported in previous studies have belonged to serogroup A. The detection of NG meningococcal isolates in apparently healthy household contacts clearly indicates the importance of vigilance through surveillance and periodic in-depth studies.

## 1. Introduction

The human nasopharyngeal mucosa is the only natural reservoir of the bacterium *Neisseria meningitides* [1].Meningococci are transferred from one person to another by inhalation of infected aerosols from a previously colonized host [2]. Following an incubation period of 2-10 days, in some individuals, meningococci can pass through the epithelial cells, enter the bloodstream and then disseminate to the brain, leading to meningitis, a disease with a high case fatality rate [3]. Rates of transmission and carriage increase in closed and semi-closed populations such as military recruits, university students, pilgrims and in the household contacts of a case of meningococcal disease [4]. Although about 10% of individuals from the general population may carry *N. meningitides* in the nasopharynx, the meningococcal carriage is known to vary in relation to the age group of the population, with low carriage rate in young children, particularly under 2 years, and high rate in teenagers and young adults [5]. Moreover, the carrier state may be chronic, lasting for several months, or intermittent or transient [6].

The relationship between carriage and its progression to invasive disease is not fully understood [7]. However, nasopharyngeal carriage is the first step to invasive infection [8]. Investigation of the carrier state may contribute significantly to the understanding of the epidemiology and pathogenesis of disease caused by *N. meningitides* [2]. Identification of age groups carrying and transmitting meningococci aids in the optimization of cost-effective and long-term control of the disease through vaccination [9].In Nepal, meningococcal vaccines have not yet been introduced into the national immunization program (NIP). Therefore, baseline information on meningococcal disease and carriage will help authorities to make evidence-based decisions about the use of meningococcal vaccines in the NIP [10]. Although most reported carriage studies have been cross-sectional, longitudinal studies are important for monitoring the length of carriage over time [11]. Hence, this study was conducted to estimate the carriage rate of meningococci among household contacts of laboratory-confirmed meningococcal meningitis cases.

## 2. Results

### 2.1. Meningococal Carriage Rate among Houshold Contacts

Among the 125 household contacts of meningococcal meningitis cases, 19 (15) from 10 different households harbored meningococci at the time of the first visit. The median age of participants was 40 years (range: 8–84 years). The meningococcal carriers had a median age of 51 years (range: 23–65 years). The highest number of meningococcal carriers were adults aged above 45 years, and this was statistically significant (*p* = 0.004). None of the participants below 19 years were meningococcal carriers. There was a slight non-significant predominance of male meningococcal carriers compared to females (*p* = 0.807) (Table 1).

The meningococcal carriage rate among household contacts was reduced from 15 during the 1st visit to 2 during the 5th visit. The duration of carriage was 60 days (Table 2). None of the participants who were identified as carriers developed meningitis within the follow-up period.

The maximum carriage duration of 60 days during the follow up study was observed in the mother (43 years old) and father (55 years old) of the meningococcal meningitis case.

### 2.2. Serogroup Distribution of Menincococcal Isolates

Out of 36 isolates from 19 carriers, 20 isolates belonged to serogroup A, and 16 were non-serogroupable (NG). Serogroup isolated from the same individual did not change throughout the study period (Table 3).

## 3. Discussion

Since asymptomatic carriers are the key source of transmission, information on meningococcal carriage in the community increases the understanding of transmission dynamics and hence, aids in planning preventive strategies for reducing transmission [12]. Household contacts of meningococcal cases have a risk of developing meningococcal disease approximately 500–800 times the age-specific incidence in the general population [13]. Hence, we selected household contacts to increase the possibility of tracking a greater number of carriers to aid our longitudinal study design.

The carriage rate of meningococci among 125 household contacts in this study (15%) was similar to other studies in Spain (16.8%) [14], the southwest of England (18.2%) [15] and New Zealand (20.4%) [16]. As the meningococcal carriage rate varies depending on the human population, it has been estimated to be 10–35% in young adults in Europe and the United States and 3–30% within the African meningitis belt [7,17]. The carriage rate of meningococci was 1.6–9.9% in low- and middle-income countries of the Americas and 1.4–14.2% in Asia [18]. A relatively lower carriage rate (0–1.4%) was reported in our region from 0 [19] to 1.4% [20]. These studies might have underestimated the actual carriage rate, as inoculation of samples into culture media was not done onsite immediately after sample collection. The detection of carriage in healthy individuals is greatly influenced by the swabbing technique and culture conditions [21]. Meningococci are extremely sensitive to drying. Swabs should be directly plated onsite into a selective medium or placed in a transport medium such as Stuart medium before being transported to the laboratory and plated immediately [22]. Identification of meningococci can be enhanced by the use of acridine orange stain [23] and PCR, in addition to conventional cultural techniques [24].

The meningococcal carriage rate is greatly influenced by age. A study in Europe reported that carriage rate was very low during the first years of life, with a peak in those aged 20–24 years [17]. The peak of carriage in the African meningitis belt has been reported at a relatively younger age (10 years) compared to Europe [25], with children younger than 5 years of age identified as carriers within a household [26]. In our study, all of the meningococcal carriers were above 19 years of age. The high carriage rate among teenagers, with a peak at 19years old in high income countries, might be related to the teenagers’ contact patterns and social behaviors including increased numbers of pub and club visits and kissing partners [27]. On the other hand, in Southeast Asian countries such as Nepal, there are restrictions and social norms for teenagers, which might partly explain the absence of carriage among children [28]. Carriage of meningococci among adults may be an efficient process of immune sensitization. On the other hand, children, who lack antibodies to pathogenic meningococci strains, developed disease rather than becoming carriers [29]. This provides a reasonable explanation for the observation that the prevalence of meningococcal disease is higher among children as compared to adults and that meningococcal carriage is higher among adults as compared to children. This study showed that carriage was slightly more common in males than in females, but this was not statistically significant, which agreed with the finding of other researchers [30]. Social behavior, rather than gender, might explain the higher frequency of meningococcal carriers among males.

Similar to our study, the average duration of carriage was 2.9 months in an urban setting in Mali and 3 months in Nigeria [12,31], while relatively shorter (30 days) and longer (6 months) durations of carriage were reported in other settings [11,32]. An older study estimated an average carriage duration of 9 months [33]. In addition to the host factors, the duration of carriage depends on the properties of the colonizing strain [22]. The longest duration of carriage observed in this study was in immediate family members, i.e.,the father and mother, the main caregivers for the meningococcal meningitis case. Monitoring of carriage duration among the same individuals over time requires longitudinal studies. However, most of the reported carriage studies are cross-sectional because of the challenges of conducting a longitudinal study, i.e., the burden of repeated swabbing of the same individuals during long-term follow-up [12]. To the best of our knowledge, this was the first longitudinal study on meningococcal carriage in Kathmandu, Nepal.

More than 90% of invasive meningococcal disease is caused by serogroups A, B, C, W and Y. Out of 19 carriers among 125 participants in our study, 11 harbored non-serogroupable meningococcal isolates. The predominance of non-serogroupable isolates in our study (9%) was in agreement with previous studies, but in contrast with the study conducted in Burkina Faso, which ranged from 1.5% [34] to 16.5% [35]. Carried strains often lack capsules and hence, are not serogroupable. The lack of capsule might be due to the deletion of the capsule locus (null or cnl) or the down regulation of the expression of the capsule. This, in turn, enhances the capability of meningococci to colonize the human nasopharynx [36]. In Nepal, it is not generally recommended to use antibiotics in the close contacts of meningococcal cases within 24 hours to eliminate the carriage of *N. meningitidis*. However, our findings indicated that substantial spread of meningococci occurred within households. Therefore, chemoprophylaxis with antibiotics should be done among asymptomatic carriers to interrupt the chain of transmission.

The major limitation of our study was that we didn’t know the status of meningococcal carriage among households contacts prior to our study. Hence, we could not conclude the maximum duration of carriage among those individuals from our study. The existence of nonserogroupable isolates indicated that further molecular characterization of the carriage strains was warranted.

## 4. Materials and Methods

### 4.1. Study Design

This longitudinal study was conducted from January 2017 to December 2019 among household contacts of laboratory-confirmed meningococcal meningitis cases. Five study sites within the Kathmandu Valley of Nepal were selected, which was due to the identification of meningococcal meningitis cases during our previous cross-sectional, multicentric, hospital-based study [37].

### 4.2. Sample Size

A few studies have revealed that the meningococcal carriage rate in Nepal has been between 0 and 1.4% [19,20].There was no published study on the rate of meningococcal carriage in household contacts in Nepal to aid the study design. Hence, we pragmatically decided that the maximum number of households included would be five per study site. Samples were collected at the first visit from each person, twice a month for upto two months, and subsequently, on a monthly basis, for a further 4 months. A maximum of nine samples was collected from each person. Altogether, 1125 throat swab samples were included from 125 persons of 25 households at five different sites. The number of family members from each household ranged from 3 to 7.

Within two days of identification of the meningococcal meningitis case, household contacts were invited to participate in this carriage study. Household contacts were defined as the family members of the case living in the same house and the persons who had close contact with a case of meningococcal disease 7 days prior to onset of symptoms to 24 h after the initiation of the appropriate antibiotic therapy [13,38].

### 4.3. Inclusion Criteria

Household contacts willing to provide repeated samples in the longitudinal study were included in the study.

### 4.4. Exclusion Criteria

Persons under antibiotics within the past 7 days were not included in the study.

### 4.5. Sample Collection and Processing

Throat samples were collected from each person by gently rubbing the uvula and tonsillar region with a sterile swab with a plastic shaft (Hi Media Laboratories, Pvt. Limited, Mumbai, India) [1]. Each sample was immediately inoculated into a modified Thayer-Martin medium (MTM) plate, which was prepared in-house using gonococci (GC) agar base (Oxoid CM0367B);hemoglobin powder (Hi Media Laboratories, Pvt. Limited, Mumbai, India); VCNT supplement, containing 3 mg/L vancomycin; 7.5 mg/L colistin methanesulfonate; 12.5 U/L nystatin; and 5 mg/L trimethoprim lactate (Sigma V0133, Bangalore, India) and Vitox enrichment supplement (Oxoid SR0090A, Waltham, MA, USA), rehydrated with Vitox rehydrate (Oxoid SR0090B, Waltham, MA, USA). Each inoculated plate was transported to the National Public Health Laboratory (NPHL), Teku, Kathmandu, Nepal within 1–2 h of collection and incubated at 37 °C in a candle jar for 18–24 h [39]. To assure quality control, at the sample collection site, one plate was swabbed with a sterile, unused, fresh swab into the MTM plate, and another plate with the *N. meningitidis* Z1503 strain, which was one of the 107 standard reference strains used in the first paper describing multilocus sequence typing [40]. On MTM, colonies of meningococci appeared as large, mucoid and bluish gray. Each suspected colony was sub-cultured on blood agar (BA) and incubated at 37 °C in a candle jar for 18–24 h. On BA, colonies appeared gray, unpigmented, round, smooth, moist, glistening and convex with a clearly defined edge. On chocolate agar (CA), colonies appeared from large and colorless to gray and opaque [41]. 

### 4.6. Species Identification 

Identification of *N. meningitidis* was done at the National Public Health Laboratory (NPHL), Teku, Kathmandu, Nepal using standard microbiological techniques including colony characteristics, Gram staining, oxidase tests and carbohydrate utilization tests [41,42]. 

### 4.7. Serogrouping

Serogrouping of *N. meningitides* isolate was done through slide agglutination testing using group-specific antisera (Remel Europe, Ltd., Dartford, UK) for serogroups A, B, C, W and Y, as described previously [37].

### 4.8. Ethical Approval 

Ethical approval for the study was obtained from the Nepal Health Research Council (Reg. No. 465/2016). Throat samples were collected only after obtaining informed consent from the participants or their parents, in the case of children.

### 4.9. Data Analysis

The obtained data were entered into Microsoft Office Excel 2007, exported to IBM SPSS Statistics 21 software and analyzed. Data on variables such as age group and gender were calculated as percentages and compared using the chi-square test. A *p*-value of <0.05 was considered statistically significant. 

## 5. Conclusions

All of the meningococcal isolates over the past 38 years in Nepal reported in previous studies belonged to serogroup A. The detection of non-serogroupable meningococcal isolates in apparently healthy household contacts clearly indicates the importance of vigilance through surveillance and periodic, in-depth studies.

## Figures and Tables

**Table 1:** Age-and gender-wise distribution of participants and asymptomatic meningococcal carriers.

Age Group	Male Number (%)		Female Number (%)		Total Number
	Participants	Meningococcal Carriers	Participants	Meningococcal Carriers	
Child (1–10 years)	4 (50)	-	4 (50)	-	8
Adolescent (10–19 years)	3 (30)	-	7 (70)	-	10
Adults (19–45 years)	26 (41)	2 (8)	38 (59)	5 (13)	64
Adults (above 45 years)	23 (53)	7 (30)	20 (47)	5 (25)	43
Total Number	56	9	69	10	125

**Table 2:** Number of asymptomatic meningococcal carriers according to the day of sample collection.

Number of Visit	Day of Sample Collection	Participants Positive for Meningococci	
		Number	Percentage
1st	0	19	15
2nd	15	8	7
3rd	30	5	4
4th	45	2	2
5th	60	2	2
6th	90	0	-
7th	120	0	-
8th	150	0	-
9th	180	0	-

**Table 3:** Serogroup distribution of meningococci isolated from asymptomatic carriers.

Carrier Number	Visit No. (Day of Throat Swab Sample Collection)								
	1st (0)	2nd (15)	3rd (30)	4th (45)	5th (60)	6th (90)	7th (120)	8th (150)	9th (180)
1	A	A	A	A	A	-	-	-	-
2	A	-	A	-	-	-	-	-	-
3	A	-	-	-	-	-	-	-	-
4	NG	NG	NG	-	-	-	-	-	-
5	NG	-	-	-	-	-	-	-	-
6	NG	-	-	-	-	-	-	-	-
7	A	-	A	-	-	-	-	-	-
8	NG	NG	-	-	-	-	-	-	-
9	NG	-	-	-	-	-	-	-	-
10	A	A	A	A	A	-	-	-	-
11	NG	NG	-	-	-	-	-	-	-
12	NG	NG	-	-	-	-	-	-	-
13	NG	-	-	-	-	-	-	-	-
14	A	A	-	-	-	-	-	-	-
15	NG	-	-	-	-	-	-	-	-
16	A	A	-	-	-	-	-	-	-
17	NG	-	-	-	-	-	-	-	-
18	A	-	-	-	-	-	-	-	-
19	NG	-	-	-	-	-	-	-	-

A: *N. meningitidis* serogroup A; NG: *N. meningitidis* non-serogroupable; symbol “-” indicates negative result.

## Data Availability

The data generated or analyzed during this study are included in this published article.
